# Smoke-Free Rules and Secondhand Smoke Exposure in Homes and Vehicles Among US Adults, 2009–2010

**DOI:** 10.5888/pcd10.120218

**Published:** 2013-05-16

**Authors:** Brian A. King, Shanta R. Dube, David M. Homa

**Affiliations:** Author Affiliations: Shanta R. Dube, David M. Homa, Centers for Disease Control and Prevention, Atlanta, Georgia.

## Abstract

**Introduction:**

An increasing number of US states and localities have implemented comprehensive policies prohibiting tobacco smoking in all indoor areas of public places and worksites. However, private settings such as homes and vehicles remain a major source of exposure to secondhand smoke (SHS) for many people. This study assessed the prevalence and correlates of voluntary smoke-free rules and SHS exposure in homes and vehicles among US adults.

**Methods:**

We obtained data from the 2009–2010 National Adult Tobacco Survey, a landline and cellular-telephone survey of adults aged 18 years or older residing in the 50 US states or the District of Columbia. We calculated national and state estimates of smoke-free rules and past-7-day SHS exposure in homes and vehicles and examined national estimates by sex, age, race/ethnicity, and education.

**Results:**

The national prevalence of voluntary smoke-free home rules was 81.1% (state range, 67.9%–92.9%), and the prevalence of household smoke-free vehicle rules was 73.6% (state range, 58.6%–85.8%). Among nonsmokers, the prevalence of SHS exposure was 6.0% in homes (state range, 2.4%–13.0%) and 9.2% in vehicles (state range, 4.8%–13.7%). SHS exposure among nonsmokers was greatest among men, younger adults, non-Hispanic blacks, and those with a lower level of education.

**Conclusion:**

Most US adults report having voluntary smoke-free home and vehicle rules; however, millions of people remain exposed to SHS in these environments. Disparities in exposure also exist among certain states and subpopulations. Efforts are needed to warn about the dangers of SHS and to promote voluntary smoke-free home and vehicle rules.

## Introduction

Secondhand smoke (SHS) is a mixture of the smoke produced by the burning end of a tobacco product and the smoke exhaled by smokers (1). Exposure to SHS causes heart disease and lung cancer in adult nonsmokers, and it causes sudden infant death syndrome, acute respiratory infections, ear problems, and more severe asthma in children (1). Each year, SHS exposure causes an estimated 3,400 deaths due to lung cancer and more than 46,000 deaths due to heart disease among US adult nonsmokers (2). The Surgeon General concluded that no risk-free level of SHS exists and that eliminating smoking in indoor spaces is the only effective way to fully protect nonsmokers from the adverse effects of SHS exposure (1,3).

In the United States, considerable progress has been made toward increasing the number of statewide comprehensive smoke-free policies that prohibit tobacco smoking in all indoor areas of public places and worksites, including restaurants and bars. As of December 2012, 26 US states and the District of Columbia have enacted comprehensive smoke-free policies (4). Such policies reduce SHS exposure and the incidence of certain adverse health events among nonsmoking hospitality workers and members of the general public (1,5,6). However, comprehensive smoke-free policies do not eliminate SHS exposure from all sources. Private settings, such as homes and vehicles, remain a major source of SHS exposure for many people (1). Nearly all nonsmokers who live with someone who smokes inside their home are exposed to SHS (7).

The percentage of US households that have voluntary smoke-free home rules increased from 43.1% in 1992–1993 to 79.1% in 2006–2007 (8), and SHS exposure in the home declined from 20.9% in 1988–1994 to 10.2% in 1999–2004 (9). However, the extent of SHS exposure and the extent to which smoke-free rules were adopted by US adults in recent years is not known, particularly at the state level (10,11). The prevalence of smoke-free rules and SHS exposure in vehicles has been assessed among some subpopulations, but no studies have examined these indicators among US adults (12,13). To reduce this gap in knowledge, we analyzed data from the 2009–2010 National Adult Tobacco Survey (NATS) to determine national and state estimates of the prevalence and sociodemographic correlates of voluntary smoke-free rules and SHS exposure in homes and vehicles among US adults.

## Methods

### Sample

The 2009–2010 NATS was a stratified, national telephone survey of noninstitutionalized adults aged 18 years or older and residing in 1 of the 50 US states or the District of Columbia. Methods for NATS are detailed elsewhere (14). In brief, the sample was designed to yield representative national and state data. Each state was divided into strata by telephone type. For the landline component, each state was allocated an equal target sample size (n = 1,863) to ensure adequate precision for state estimates. For the cellular-telephone component, each state was allocated a sample size in proportion to its population (range, 255–24,100). Louisiana, New Jersey, and Oklahoma added to their landline and cellular-telephone target sample size; Delaware, Georgia, Iowa, North Dakota, Pennsylvania, South Carolina, and Virginia added to their landline target sample size.

Respondent selection varied by telephone type. For landline numbers, 1 adult was randomly selected from each eligible household. For cellular-telephone numbers, the adult reached was selected if the cellular telephone was the only way the adult could be reached by telephone at home. In total, 118,581 interviews were completed (110,634 landline interviews; 7,947 cellular-telephone interviews) from October 2009 through February 2010. The Council of American Survey and Research Organizations (CASRO) response rate (15), defined as the number of completed interviews divided by the number of eligible respondents in the sample, was 37.6% (landline, 40.4%; cellular, 24.9%). The national cooperation rate, defined by CASRO as the number of completed interviews divided by the number of eligible respondents who were successfully reached by an interviewer, was 62.3% (landline, 61.9%; cellular, 68.7%). State CASRO response rates ranged from 28.2% in New Jersey to 49.3% in Vermont (median, 37.9%); state cooperation rates ranged from 52.9% in Louisiana to 72.4% in Vermont (median, 62.9%).

### Measures

The presence of smoke-free home rules was determined by answers to the question, “Not counting decks, porches, or garages, inside your home, is smoking ‘always allowed,’ ‘allowed only at some time or in some places,’ or ‘never allowed’?” Respondents who answered “never allowed” were classified as having a smoke-free home rule. Having a smoke-free vehicle rule was determined by answers to the question, “Not counting motorcycles, in the vehicles that you or family members who live with you own or lease, is smoking ‘always allowed in all vehicles,’ ‘sometimes allowed in at least one vehicle,’ or ‘never allowed in any vehicle’?” Respondents who answered “never allowed in any vehicle” were classified as having a household smoke-free vehicle rule.

Exposure to SHS in the home was determined by answers to the question, “Not counting decks, porches, or garages, during the past 7 days, on how many days did someone other than you smoke tobacco inside your home while you were at home?” Open response options ranged from 0 to 7. Respondents who answered from 1 through 7 were classified as exposed to SHS in their home within the previous 7 days. Exposure to SHS in a vehicle was determined by answers to the question, “During the past 7 days, on how many days did you ride in a vehicle where someone other than you was smoking tobacco?” Open response options ranged from 0 through 7. Respondents who answered from 1 through 7 were classified as exposed to SHS in a vehicle within the previous 7 days.

Smoking status was determined by answers to the questions, “Have you smoked at least 100 cigarettes in your entire life?” and “Do you now smoke cigarettes every day, some days, or not at all?” Respondents who reported smoking at least 100 cigarettes in their lifetime and who indicated they now smoke “every day” or “some days” were classified as current smokers. Respondents who reported not smoking 100 cigarettes in their lifetime or who reported smoking at least 100 cigarettes in their lifetime but now smoke cigarettes “not at all” were classified as nonsmokers. Sample size constraints prevented our analyzing former and never-smokers separately.

We examined the following characteristics: sex, age (18–24, 25–44, 45–64, or ≥65 years) race/ethnicity (non-Hispanic white, non-Hispanic black, Hispanic, non-Hispanic Asian, or non-Hispanic other), and education (0–12 years [no diploma], graduate equivalency degree [GED], high school graduate, some college [no degree], associate degree, undergraduate degree, or graduate degree). For race/ethnicity, “non-Hispanic other” were respondents who were American Indian or Alaska Native, Native Hawaiian or Pacific Islander, multiracial, or some other race. For annual household income, respondents were asked about the combined annual income from all sources for every person living in their household; of all respondents, 11.9% did not answer the question.

### Data analysis

We analyzed data by using SAS-callable SUDAAN version 9.2 (RTI International, Research Triangle Park, North Carolina). The landline data were first weighted by the inverse of the probability of selection of the telephone number, a nonresponse adjustment, and adjustments for number of landlines and number of eligible subjects in a household. The cellular-telephone data were first weighted by the inverse of the probability of selection of the telephone number and a nonresponse adjustment. Next, the data were poststratified by state according to the distribution of demographic variables (sex, age, race/ethnicity, marital status, and educational attainment) and telephone type. For states with a small number of cellular-telephone respondents, the use of both landline and cellular-telephone data resulted in a large unequal weighting effect and, therefore, large estimated variances of survey estimates and small effective sample sizes. As a result, we calculated national and state estimates differently. For national estimates, we included both cellular-telephone and landline respondents. For state estimates, we included cellular-telephone respondents only for the 12 states that had a cellular-telephone sample of 200 or more (California, Florida, Georgia, Illinois, Louisiana, New Jersey, New York, North Carolina, Ohio, Oklahoma, Pennsylvania, and Texas). Differences between estimates were considered statistically significant if 95% confidence intervals did not overlap.

## Results

The overall percentage of respondents with a smoke-free home rule was 81.1%; the prevalence was significantly higher among nonsmokers (89.1%) than among smokers (48.0%) (Table 1). The overall prevalence of smoke-free home rules was significantly higher among women (82.6%) than among men (79.5%); however, among smokers, men were significantly more likely to have such rules. By age, overall prevalence of a smoke-free home rule was highest among those aged 25 to 44 (83.2%) and 65 years old or older (83.2%); among smokers, prevalence of a rule decreased with increasing age. By race/ethnicity, prevalence of a smoke-free home rule was highest among non-Hispanic Asians (90.6%) and Hispanics (87.7%) and lowest among non-Hispanic blacks (73.8%). By education, prevalence was lowest among adults with a GED (63.6%) (Table 1). By state, prevalence ranged from 67.9% in Kentucky to 92.9% in Utah (Table 2).

**Table 1:** Percentage (95% CI) of Adults Who Reported Having 100% Smoke-Free Home and Vehicle Rules, by Smoking Status and Selected Characteristics, National Adult Tobacco Survey, 2009–2010^a^

Characteristic	100% Smoke-Free Home Rule^b^			100% Smoke-Free Vehicle Rule^c^		
	Nonsmokers (n = 101, 073)	Smokers (n = 16,497)	Overall^d^ (n = 117,999)	Nonsmokers (n = 100,284)	Smokers (n = 16,213)	Overall^e^ (n = 116,914)
**Sex**						
Male	87.7 (87.0–88.4)	51.1 (48.9–53.3)	79.5 (78.7–80.3)	83.3 (82.4–84.1)	27.8 (25.7–30.0)	70.9 (70.0–71.8)
Female	90.4 (89.9–90.9)	44.2 (42.2–46.2)	82.6 (82.0–83.2)	86.3 (85.7–86.9)	25.9 (24.2–27.8)	76.2 (75.5–76.9)
**Age, y**						
18–24	83.7 (81.6–85.5)	59.2 (54.9–63.5)	77.9 (76.0–79.7)	72.2 (69.9–74.4)	24.9 (21.0–29.3)	61.1 (59.0–63.2)
25–44	91.8 (91.1–92.4)	54.7 (52.3–57.2)	83.2 (82.3–84.0)	85.7 (84.8–86.6)	27.5 (25.2–30.0)	72.3 (71.3–73.3)
45–64	89.2 (88.5–89.8)	37.0 (35.0–39.2)	79.0 (78.2–79.7)	86.4 (85.7–87.0)	25.5 (23.7–27.4)	74.6 (73.8–75.4)
≥65	87.9 (87.1–88.6)	34.0 (29.1–39.3)	83.2 (82.3–84.0)	89.2 (88.5–89.9)	35.1 (30.7–39.8)	84.7 (83.8–85.6)
**Race/ethnicity**						
White, non-Hispanic	89.3 (88.9–89.7)	46.8 (45.2–48.4)	80.9 (80.4–81.4)	84.8 (84.3–85.3)	22.5 (21.2–23.9)	72.6 (72.0–73.1)
Black, non-Hispanic	84.3 (82.5–86.0)	34.6 (30.3–39.1)	73.8 (71.9–75.6)	81.9 (80.0–83.8)	32.9 (28.8–37.3)	71.7 (69.7–73.6)
Hispanic	92.2 (90.6–93.6)	67.4 (61.2–73.1)	87.7 (86.0–89.3)	86.3 (84.1–88.2)	46.0 (39.5–52.7)	79.1 (76.8–81.3)
Asian, non-Hispanic	92.3 (89.3–94.5)	66.1 (51.6–78.1)	90.6 (87.7–92.9)	93.1 (90.6–95.0)	46.3 (31.0–62.3)	90.0 (87.2–92.2)
Other, non-Hispanic	86.0 (82.6–88.8)	48.2 (42.4–54.1)	75.4 (72.5–78.1)	81.9 (78.5–84.8)	23.1 (18.2–28.9)	65.7 (62.6–68.8)
**Education**						
0–12 years (no diploma)	84.9 (83.0–86.7)	40.9 (36.9–45.0)	71.3 (69.3–73.3)	82.1 (79.9–84.1)	28.7 (25.1–32.7)	65.8 (63.5–68.0)
GED	83.3 (79.1–86.7)	40.9 (34.5–47.7)	63.6 (59.7–67.4)	76.9 (72.1–81.1)	17.5 (13.1–23.1)	49.1 (44.9–53.2)
High school graduate	86.0 (85.0–87.0)	47.9 (45.3–50.6)	77.6 (76.6–78.6)	81.4 (80.2–82.5)	26.0 (23.6–28.4)	69.1 (68.0–70.3)
Some college (no degree)	89.4 (88.4–90.3)	50.5 (47.4–53.7)	81.8 (80.7–82.8)	82.7 (81.4–83.9)	24.8 (22.1–27.8)	71.5 (70.2–72.7)
Associate degree	90.7 (89.7–91.5)	52.9 (49.4–56.3)	83.4 (82.3–84.4)	85.4 (84.2–86.5)	27.3 (24.0–30.9)	74.1 (72.8–75.4)
Undergraduate degree	93.0 (92.3–93.6)	62.1 (58.5–65.7)	90.3 (89.6–91.0)	89.6 (88.9–90.3)	33.7 (30.0–37.6)	84.8 (84.0–85.6)
Graduate degree	93.8 (93.1–94.4)	53.4 (47.7–58.9)	91.5 (90.8–92.2)	92.0 (91.2–92.7)	35.1 (29.9–40.6)	88.8 (88.0–89.6)
**All**	89.1 (88.7–89.5)	48.0 (46.5–49.5)	81.1 (80.6–81.6)	84.9 (84.4–85.4)	27.0 (25.6–28.4)	73.6 (73.1–74.2)

Abbreviations: CI, confidence interval; GED, graduate equivalency degree.

a All estimates were calculated among both landline and cellular-telephone respondents.

b The presence of smoke-free home rules was determined by answers to the question, “Not counting decks, porches, or garages, inside your home, is smoking ‘always allowed,’ ‘allowed only at some time or in some places,’ or ‘never allowed’?” Respondents who answered “never allowed” were classified as having a 100% smoke-free home rule.

c The presence of a household smoke-free vehicle rule was determined by answers to the question, “Not counting motorcycles, in the vehicles that you or family members who live with you own or lease, is smoking ‘always allowed in all vehicles,’ ‘sometimes allowed in at least one vehicle,’ or ‘never allowed in any vehicle’?” Respondents who answered “never allowed in any vehicle” were classified as having a 100% smoke-free vehicle rule.

d Includes 429 respondents whose smoking status was unknown.

e Includes 417 respondents whose smoking status was unknown.

**Table 2:** Percentage (95% CI) of Adults Who Reported Having 100% Smoke-Free Home and Vehicle Rules, by State, National Adult Tobacco Survey, 2009–2010

State	100% Smoke-Free Home Rule^a^		100% Smoke-Free Vehicle Rule b	
	n	% (95% CI)	n	% (95% CI)
Alabama	1,885	75.7 (72.3–78.8)	1,870	70.0 (66.4–73.4)
Alaska	1,834	84.9 (82.0–87.5)	1,799	72.1 (68.6–75.4)
Arizona	1,789	86.2 (82.9–89.0)	1,783	82.7 (79.4–85.6)
Arkansas	2,800	75.7 (72.9–78.3)	2,778	69.0 (65.9–71.9)
California^c^	2,558	90.1 (88.4–91.6)	2,537	83.8 (81.6–85.7)
Colorado	1,806	85.9 (83.0–88.3)	1,791	75.9 (72.3–79.1)
Connecticut	1,833	84.2 (80.9–87.1)	1,818	77.2 (73.3–80.7)
Delaware	1,960	82.0 (79.1–84.7)	1,939	71.8 (68.4–75.1)
District of Columbia	1,852	76.7 (72.6–80.4)	1,793	75.3 (69.7–80.2)
Florida^c^	2,257	85.8 (83.6–87.8)	2,247	75.1 (72.2–77.7)
Georgia^c^	4,899	82.9 (80.8–84.9)	4,853	75.6 (73.3–77.7)
Hawaii	1,776	80.3 (76.8–83.3)	1,747	78.4 (74.8–81.7)
Idaho	1,765	91.5 (89.6–93.2)	1,759	79.8 (76.2–83.0)
Illinois^c^	2,043	77.0 (74.2–79.6)	2,026	71.4 (68.4–74.3)
Indiana	1,868	76.2 (72.9–79.2)	1,855	65.7 (62.1–69.0)
Iowa	2,037	80.8 (77.8–83.4)	2,019	71.4 (67.7–74.9)
Kansas	1,838	79.4 (76.3–82.2)	1,827	72.2 (68.7–75.4)
Kentucky	1,764	67.9 (64.2–71.4)	1,746	58.6 (54.7–62.4)
Louisiana^c^	6,335	77.2 (75.2–79.1)	6,254	69.7 (67.6–71.8)
Maine	1,986	84.4 (82.1–86.5)	1,965	73.1 (70.0–76.0)
Maryland	1,828	84.8 (81.7–87.5)	1,819	76.7 (72.5–80.4)
Massachusetts	1,804	80.1 (76.1–83.5)	1,781	73.9 (69.7–77.7)
Michigan	1,813	76.7 (73.4–79.8)	1,797	67.0 (63.3–70.6)
Minnesota	1,778	85.4 (82.8–87.7)	1,760	76.4 (73.0–79.5)
Mississippi	1,747	75.8 (71.9–79.3)	1,724	66.6 (62.2–70.8)
Missouri	1,853	74.3 (70.7–77.6)	1,836	67.3 (63.7–70.7)
Montana	1,816	86.0 (83.3–88.2)	1,809	74.1 (70.3–77.5)
Nebraska	1,820	83.5 (80.5–86.2)	1,813	70.4 (66.5–74.0)
Nevada	1,795	82.6 (79.6–85.2)	1,780	72.0 (68.1–75.6)
New Hampshire	1,925	84.8 (82.0–87.3)	1,906	76.9 (73.7–79.8)
New Jersey^c^	4,077	82.9 (80.9–84.8)	4,050	77.2 (75.0–79.3)
New Mexico	1,773	83.2 (79.9–86.0)	1,764	78.4 (74.7–81.7)
New York^c^	2,226	80.1 (77.4–82.6)	2,168	77.0 (74.1–79.6)
North Carolina^c^	2,011	75.5 (72.5–78.3)	2,000	69.3 (66.2–72.3)
North Dakota	2,182	84.2 (81.4–86.6)	2,172	75.5 (72.1–78.5)
Ohio^c^	2,137	71.9 (69.4–74.3)	2,110	65.3 (62.6–67.8)
Oklahoma^c^	3,635	74.1 (72.2–75.9)	3,612	65.4 (63.4–67.4)
Oregon	1,858	89.6 (86.4–92.0)	1,839	79.0 (75.1–82.5)
Pennsylvania^c^	3,408	76.8 (74.7–78.7)	3,374	71.3 (69.2–73.3)
Rhode Island	1,891	81.8 (78.6–84.6)	1,879	73.3 (69.7–76.6)
South Carolina	5,047	79.7 (77.5–81.8)	4,995	71.8 (69.4–74.2)
South Dakota	1,984	83.9 (81.3–86.2)	1,967	74.8 (71.5–77.8)
Tennessee	1,825	74.6 (70.9–78.0)	1,811	69.8 (66.0–73.2)
Texas^c^	2,346	83.4 (81.0–85.5)	2,337	75.0 (72.2–77.5)
Utah	2,021	92.9 (90.8–94.6)	2,010	85.8 (82.6–88.5)
Vermont	2,028	80.6 (77.7–83.2)	2,015	73.1 (69.9–76.1)
Virginia	2,261	82.1 (79.2–84.6)	2,238	76.1 (73.0–78.9)
Washington	1,840	88.4 (86.0–90.5)	1,822	77.8 (74.0–81.1)
West Virginia	1,763	68.7 (65.3–72.0)	1,738	61.4 (57.7–65.0)
Wisconsin	1,821	80.9 (77.8–83.6)	1,810	73.1 (69.7–76.3)
Wyoming	1,720	81.9 (79.2–84.4)	1,714	74.5 (71.0–77.7)

Abbreviation: CI, confidence interval.

a The presence of smoke-free home rules was determined by answers to the question, “Not counting decks, porches, or garages, inside your home, is smoking ‘always allowed,’ ‘allowed only at some time or in some places,’ or ‘never allowed’?” Respondents who answered “never allowed” were classified as having a 100% smoke-free home rule.

b The presence of a household smoke-free vehicle rule was determined by answers to the question, “Not counting motorcycles, in the vehicles that you or family members who live with you own or lease, is smoking ‘always allowed in all vehicles,’ ‘sometimes allowed in at least one vehicle,’ or ‘never allowed in any vehicle’?” Respondents who answered “never allowed in any vehicle” were classified as having a 100% smoke-free vehicle rule.

c Estimates calculated among both landline and cellular-telephone respondents. All other state estimates were calculated among landline respondents only.

The overall percentage of respondents with a household smoke-free vehicle rule was 73.6%; prevalence was significantly higher among nonsmokers (84.9%) than among smokers (27.0%) (Table 1). Overall prevalence was significantly higher among women (76.2%) than among men (70.9%); among smokers, we observed no significant differences in prevalence by sex. Overall prevalence of a smoke-free vehicle rule increased with increasing age and levels of education; people with a GED had the lowest prevalence (49.1%). By race/ethnicity, smoke-free vehicle rule prevalence was highest among non-Hispanic Asians (90.0%) and lowest among non-Hispanic adults of other races (65.7%) (Table 1). By state, the prevalence of smoke-free vehicle rules ranged from 58.6% in Kentucky to 85.8% in Utah (Table 2).

Of nonsmokers, 6.0% (which extrapolates to 10.9 million US adults) were exposed to SHS in their home in the previous 7 days (Table 3); 2.0% were exposed 1 or 2 days, 0.6% were exposed 3 or 4 days, 0.3% were exposed 5 or 6 days, and 3.1% were exposed every day. Exposure was significantly lower among those with a 100% smoke-free home rule (1.4%) than among those with no rule (44.0%). Overall exposure was significantly higher among men (6.6%) than among women (5.5%); among those with no home rule, we observed no significant difference by sex. By age, exposure was highest among those aged 18 to 24 (12.7%) and lowest among those aged 65 or older (3.8%). By race/ethnicity, exposure was highest among non-Hispanic blacks (11.4%) and lowest among non-Hispanic Asians (3.0%). Exposure decreased with increasing levels of education (Table 3). By state, exposure ranged from 2.4% in Utah to 13.0% in West Virginia (Table 4).

**Table 3:** Percentage (95% CI) of Nonsmoking Adults Who Reported Exposure to Secondhand Smoke in Their Home or a Vehicle in Which They Rode During the Previous 7 Days, by Smoke-Free Rule Status and Selected Characteristics, National Adult Tobacco Survey, 2009–2010^a^

Characteristic	Secondhand Smoke Exposure in Home^b^			Secondhand Smoke Exposure in Vehicle^c^		
	Smoke-Free Home Rule (n = 91,273)	No Smoke-Free Home Rule (n = 9,591)	Overall^d^ (n = 101,370)	Smoke-Free Vehicle Rule (n = 88,291)	No Smoke-Free Vehicle Rule (n = 11,845)	Overall^e^ (n = 101,416)
**Sex**						
Male	1.4 (1.1–1.7)	43.9 (40.7–47.1)	6.6 (6.0–7.2)	5.3 (4.7–5.9)	37.6 (34.9–40.4)	10.7 (10.0–11.5)
Female	1.4 (1.2–1.6)	44.2 (41.5–46.9)	5.5 (5.1–5.9)	3.6 (3.2–4.0)	34.7 (32.5–37.0)	7.9 (7.4–8.4)
**Age, y**						
18–24	2.9 (2.0–4.0)	63.6 (56.8–69.9)	12.7 (11.0–14.5)	11.0 (9.4–12.9)	48.6 (43.8–53.4)	21.6 (19.6–23.7)
25–44	1.1 (0.9–1.4)	49.7 (45.5–54.0)	5.1 (4.6–5.7)	4.9 (4.3–5.6)	37.1 (33.9–40.4)	9.5 (8.8–10.3)
45–64	1.4 (1.1–1.7)	41.1 (38.1–44.1)	5.7 (5.2–6.2)	3.2 (2.8–3.7)	32.1 (29.6–34.7)	7.2 (6.6–7.8)
≥65	1.0 (0.8–1.3)	24.0 (21.3–26.9)	3.8 (3.4–4.2)	1.9 (1.6–2.2)	22.0 (19.1–25.2)	4.0 (3.6–4.5)
**Race/ethnicity**						
White, non-Hispanic	1.1 (0.9–1.2)	40.9 (38.7–43.1)	5.3 (4.9–5.6)	3.4 (3.1–3.7)	35.2 (33.4–37.0)	8.2 (7.8–8.7)
Black, non-Hispanic	2.9 (2.0–4.1)	58.3 (52.0–64.4)	11.4 (10.0–13.0)	6.7 (5.5–8.1)	44.7 (38.9–50.6)	13.6 (12.0–15.3)
Hispanic	1.7 (1.1–2.5)	47.8 (38.2–57.5)	5.3 (4.2–6.6)	7.4 (5.9–9.2)	34.8 (27.8–42.6)	11.1 (9.5–13.1)
Asian, non-Hispanic	0.9 (0.4–1.7)	29.5 (16.0–47.9)	3.0 (1.8–5.1)	2.6 (1.4–4.9)	38.0 (22.9–55.9)	5.0 (3.2–7.8)
Other, non-Hispanic	3.1 (1.5–6.0)	41.1 (30.6–52.5)	8.4 (6.3–11.0)	6.5 (4.6–9.1)	32.3 (25.1–40.4)	11.4 (9.2–13.9)
**Education**						
0–12 years (no diploma)	2.8 (2.0–4.0)	51.2 (44.8–57.6)	10.1 (8.7–11.8)	8.4 (6.7–10.5)	44.7 (38.5–51.1)	15.0 (13.1–17.1)
GED	3.2 (1.6–6.2)	55.5 (43.4–67.0)	11.8 (8.7–15.9)	9.2 (5.7–14.4)	51.1 (40.0–62.1)	19.1 (14.9–24.0)
High school graduate	1.6 (1.3–2.1)	48.4 (44.6–52.2)	8.1 (7.3–8.9)	5.3 (4.6–6.0)	40.2 (37.0–43.6)	11.8 (10.9–12.7)
Some college (no degree)	1.3 (1.0–1.8)	51.1 (46.4–55.8)	6.5 (5.8–7.4)	4.2 (3.6–5.1)	37.4 (33.6–41.3)	10.0 (9.0–11.0)
Associate degree	1.1 (0.8–1.5)	37.8 (33.1–42.7)	4.5 (3.9–5.1)	4.1 (3.5–4.9)	33.0 (29.1–37.1)	8.3 (7.5–9.3)
Undergraduate degree	0.8 (0.6–1.1)	26.0 (22.1–30.3)	2.6 (2.2–3.0)	2.3 (1.9–2.8)	23.0 (20.1–26.3)	4.5 (4.0–5.0)
Graduate degree	0.5 (0.4–0.8)	22.8 (17.9–28.5)	1.9 (1.5–2.4)	1.4 (1.0–1.8)	18.2 (15.0–21.8)	2.7 (2.3–3.2)
**All**	1.4 (1.2–1.6)	44.0 (41.9–46.1)	6.0 (5.7–6.3)	4.4 (4.0–4.7)	36.2 (34.4–38.0)	9.2 (8.8–9.6)

Abbreviations: CI, confidence interval; GED, graduate equivalency degree.

a All estimates were calculated among both landline and cellular-telephone respondents.

b Defined as a response between 1 and 7 to the question, “Not counting decks, porches, or garages, during the past 7 days, on how many days did someone other than you smoke tobacco inside your home while you were at home?”

c Defined as a response between 1 and 7 to the question, “During the past 7 days, on how many days did you ride in a vehicle where someone other than you was smoking tobacco?”

d Includes 506 respondents whose home smoking rule was unknown.

e Includes 1,280 respondents whose household vehicle smoking rule was unknown or whose family does not own or lease a vehicle.

**Table 4:** Percentage (95% CI) of Nonsmoking Adults Who Reported Exposure to Secondhand Smoke in Their Home or a Vehicle in Which They Rode During the Past 7 Days, by State, National Adult Tobacco Survey, 2009–2010

Characteristic	Secondhand Smoke Exposure in Home^a^		Secondhand Smoke Exposure in Vehicle^b^	
	n	% (95% CI)	n	% (95% CI)
Alabama	1,586	7.5 (5.4–10.3)	1,590	10.8 (8.3–14.1)
Alaska	1,548	5.9 (3.7–9.2)	1,550	7.8 (6.0–10.2)
Arizona	1,587	4.5 (2.5–8.0)	1,588	6.8 (4.2–10.7)
Arkansas	2,317	6.3 (4.7–8.3)	2,319	8.9 (6.9–11.4)
California^c^	2,239	2.8 (2.0–3.9)	2,238	5.5 (4.2–7.0)
Colorado	1,614	3.8 (2.5–5.9)	1,617	8.6 (6.2–11.9)
Connecticut	1,635	4.5 (3.1–6.6)	1,635	7.1 (5.0–10.0)
Delaware	1,693	6.4 (4.5–9.1)	1,691	8.6 (6.4–11.4)
District of Columbia	1,644	7.3 (4.6–11.4)	1,642	9.3 (6.0–14.1)
Florida^c^	1,942	5.2 (3.8–7.1)	1,946	11.4 (9.3–13.8)
Georgia^c^	4,222	4.7 (3.6–5.9)	4,221	10.7 (8.8–12.9)
Hawaii	1,580	4.0 (2.7–5.9)	1,578	5.7 (3.8–8.4)
Idaho	1,583	4.3 (3.0–6.2)	1,583	6.6 (4.2–10.2)
Illinois^c^	1,778	7.0 (5.3–9.2)	1,780	10.8 (8.5–13.6)
Indiana	1,569	6.5 (4.6–8.9)	1,570	10.4 (8.0–13.3)
Iowa	1,801	7.7 (5.3–11.0)	1,802	12.9 (9.9–16.8)
Kansas	1,612	6.0 (4.1–8.7)	1,613	12.0 (9.2–15.4)
Kentucky	1,420	9.7 (7.2–13.0)	1,422	11.6 (8.7–15.5)
Louisiana^c^	5,213	8.3 (6.8–10.0)	5,218	13.5 (11.7–15.5)
Maine	1,725	3.4 (2.4–4.8)	1,725	9.6 (7.4–12.4)
Maryland	1,651	4.8 (3.2–7.2)	1,649	8.6 (6.4–11.6)
Massachusetts	1,631	5.8 (3.9–8.4)	1,629	8.8 (6.3–12.2)
Michigan	1,577	8.1 (5.9–11.1)	1,579	11.3 (8.3–15.0)
Minnesota	1,581	3.7 (2.4–5.6)	1,579	8.4 (6.2–11.2)
Mississippi	1,485	7.3 (5.0–10.4)	1,483	9.0 (6.7–11.9)
Missouri	1,566	8.7 (6.4–11.6)	1,567	11.7 (9.1–15.0)
Montana	1,618	4.6 (3.0–7.0)	1,617	6.0 (4.2–8.6)
Nebraska	1,595	5.7 (3.5–9.2)	1,597	11.6 (8.5–15.7)
Nevada	1,469	4.8 (3.2–7.0)	1,472	11.3 (8.1–15.5)
New Hampshire	1,716	4.4 (2.9–6.6)	1,716	9.8 (7.4–13.0)
New Jersey^c^	3,534	6.5 (5.3–8.1)	3,541	7.3 (6.1–8.7)
New Mexico	1,558	5.3 (3.5–7.9)	1,558	8.2 (5.7–11.7)
New York^c^	1,922	4.1 (3.0–5.7)	1,928	8.8 (6.9–11.2)
North Carolina^c^	1,706	9.5 (7.2–12.3)	1,704	10.2 (8.1–12.8)
North Dakota	1,904	3.3 (2.0–5.4)	1,905	7.4 (5.1–10.7)
Ohio^c^	1,766	8.9 (7.2–10.9)	1,765	10.7 (8.8–12.9)
Oklahoma^c^	2,870	8.8 (7.4–10.4)	2,871	11.8 (10.2–13.7)
Oregon	1,650	3.6 (1.9–6.6)	1,651	4.8 (2.9–7.9)
Pennsylvania^c^	2,896	7.9 (6.5–9.5)	2,895	8.3 (6.9–10.0)
Rhode Island	1,641	4.5 (2.9–6.8)	1,643	9.1 (6.7–12.2)
South Carolina	4,297	6.8 (5.2–9.0)	4,298	12.5 (10.3–15.3)
South Dakota	1,747	3.5 (2.4–5.2)	1,747	7.4 (5.6–9.8)
Tennessee	1,534	8.3 (5.9–11.4)	1,540	12.3 (9.6–15.8)
Texas^c^	2,003	6.3 (4.8–8.4)	2,001	9.5 (7.6–11.8)
Utah	1,896	2.4 (1.4–4.2)	1,897	6.1 (4.0–9.2)
Vermont	1,796	5.8 (4.1–8.2)	1,794	9.9 (7.5–12.9)
Virginia	1,999	4.8 (3.3–6.8)	1,999	8.3 (6.1–11.0)
Washington	1,609	3.0 (1.9–4.7)	1,612	7.1 (5.1–9.6)
West Virginia	1,441	13.0 (9.9–16.9)	1,441	13.7 (10.5–17.8)
Wisconsin	1,616	7.0 (5.1–9.5)	1,616	8.8 (6.6–11.5)
Wyoming	1,483	4.0 (2.8–5.7)	1,486	8.8 (6.1–12.5)

Abbreviation: CI, confidence interval.

a Defined as a response between 1 and 7 to the question, “Not counting decks, porches, or garages, during the past 7 days, on how many days did someone other than you smoke tobacco inside your home while you were at home?”

b Defined as a response between 1 and 7 to the question, “During the past 7 days, on how many days did you ride in a vehicle where someone other than you was smoking tobacco?”

c Estimate calculated among both landline and cellular-telephone respondents. All other state estimates were calculated among landline respondents only.

Of nonsmokers, 9.2% (which extrapolates to 16.7 million US adults) were exposed to SHS in a vehicle within the previous 7 days (Table 3); 5.9% were exposed 1 or 2 days, 1.5% were exposed 3 or 4 days, 0.5% were exposed 5 or 6 days, and 1.3% were exposed every day. Exposure was significantly lower among those with a household smoke-free vehicle rule (4.4%) than among those without such a rule (36.2%). Overall, exposure was significantly higher among men (10.7%) than among women (7.9%). By age, exposure was highest among those aged 18 to 24 (21.6%) and lowest among those aged 65 or older (4.0%). By race/ethnicity, exposure was highest among non-Hispanic blacks (13.6%) and lowest among non-Hispanic Asians (5.0%). Exposure decreased with increasing levels of education (Table 3). By state, exposure ranged from 4.8% in Oregon to 13.7% in West Virginia (Table 4).

## Discussion

This study used national and state representative samples of US adults to assess the prevalence and sociodemographic correlates of voluntary smoke-free rules and SHS exposure in private settings during 2009–2010. The findings indicate that most adults are protected by voluntary smoke-free rules in their homes and household vehicles. However, 6% of adult nonsmokers (or an estimated 10.9 million US adults) reported they were exposed to SHS in their home within the previous 7 days, and 9.2% (or an estimated 16.7 million US adults) indicated that they were exposed to SHS in a vehicle during the same period. Moreover, we observed disparities in the prevalence of smoke-free rules and SHS exposure among states and subpopulations. The implications of these findings are twofold: 1) considerable progress has been made in protecting US adults from SHS exposure in homes and vehicles through the implementation of voluntary smoke-free rules; however, 2) enhanced and sustained efforts are needed to increase awareness of the dangers of SHS exposure and to encourage the adoption of voluntary smoke-free rules in private environments, particularly among those in the subpopulations at greatest risk for SHS exposure.

The national estimate of smoke-free home rules found by this study (81.1%) was higher than previously reported estimates from 1992–1993 (43.1%) and 2006–2007 (79.1%) (8), and the estimate of home SHS exposure (6.0%) was lower than estimates from 1988–1994 (20.9%) and 1999–2004 (10.2%) (9). These encouraging changes are consistent with previously reported trends (8–10) and are probably attributable to many factors, including the proliferation of comprehensive policies prohibiting smoking inside all public places and worksites, declines in smoking prevalence, and changes in public attitudes about the social acceptability of smoking near nonsmokers and children (1). For example, we observed a higher prevalence of smoke-free rules in states such as California and Utah, which have a long history of smoke-free laws and low rates of adult smoking (16,17). This finding is consistent with study findings that show smoke-free policies in public settings stimulate the adoption of voluntary smoke-free rules in private settings (18), increase support for smoke-free environments (19), and are more strongly favored by nonsmokers than smokers (20).

Although estimates of voluntary smoke-free rules and SHS exposure found by this study are encouraging, disparities remain. The prevalence of smoke-free rules was generally higher among women than among men, older individuals, Hispanics and non-Hispanic Asians, and individuals with more education. These findings may be due to the lower rates of cigarette smoking among these groups, cultural factors related to the social disapproval of smoking, or differences in receptivity toward tobacco-related health messages and understanding of the health hazards associated with SHS exposure (17,21). To reduce these disparities, prevention efforts are needed to reach and educate all populations about the adverse health effects of SHS and to promote the voluntary adoption of smoke-free rules, particularly among subpopulations at greatest risk. For example, the US Environmental Protection Agency conducts a national campaign that educates and encourages the adoption of voluntary smoke-free rules (22). In addition, the US Public Health Service recommends that clinicians ask all patients and parents of pediatric patients whether they smoke, advise them about the dangers of SHS, and offer encouragement and help quitting (23).

The extent of SHS exposure in homes and vehicles was markedly lower among respondents protected by voluntary 100% smoke-free rules. This finding is consistent with environmental studies showing that smoke-free homes and vehicles have substantially lower levels of SHS constituents than do those in which smoking is permitted (24,25). In addition to reducing SHS exposure, research suggests that smoke-free homes can increase cessation among adult smokers and prevent relapse among former smokers (26).

An increasing number of state and local municipalities are enacting legislative policies to restrict smoking in homes and vehicles under certain conditions. For example, numerous communities in California have adopted ordinances prohibiting smoking in all living units and shared spaces of certain types of multiunit housing (27). At least 230 public housing authorities in the United States have also instituted 100% smoke-free policies in multiunit housing (28). In addition, Arkansas, California, Louisiana, Maine, and the US territory of Puerto Rico have instituted policies that prohibit smoking in vehicles occupied by children or adolescents younger than a specified age (29). However, given the greater population-level protection afforded by smoke-free policies in worksites and public places, smoke-free vehicle and multiunit housing policies are best suited for consideration following the implementation of comprehensive smoke-free policies in all public places and worksites, including restaurants and bars.

Strengths of this study include the use of recent national and state representative data and the inclusion of cellular-telephone respondents. Nonetheless, the study has some limitations. First, to prevent large variances of survey estimates and small effective sample sizes, we excluded cellular-telephone respondents from state estimates for states that had fewer than 200 cellular-telephone respondents. However, we included cellular-telephone respondents in all national estimates and in state estimates for the 12 states that had a sufficient sample size. Second, the NATS sampling frame did not include institutionalized populations or military personnel; therefore, the findings are not generalizable to these subpopulations. Third, both the limited recall period and the use of a self-reported assessment of SHS exposure could have resulted in an underestimation of true SHS exposure (30). Fourth, the NATS questionnaire addressed only home exposure to SHS that originated from smokers within the same household; SHS exposure in partially enclosed areas (eg, decks, porches, garages) and SHS exposure from neighboring units in multiunit housing were not included. Finally, the overall CASRO response rate for NATS was 37.6%, and state response rates ranged from 28.2% to 49.3%. Low response rates increase the potential for bias; however, prevalence estimates of smoke-free rules and SHS exposure in our study were comparable with estimates found by other population-level surveys with higher response rates (8–11).

In conclusion, most US adults are protected by voluntary 100% smoke-free rules in their homes and household vehicles. Nonetheless, an estimated 10.9 million adult nonsmokers remain exposed to SHS in their homes, and 16.7 million remain exposed in vehicles. Disparities in the prevalence of smoke-free rules and SHS exposure also remain among certain states and subpopulations. Because the implementation of 100% smoke-free policies is the only effective way to fully eliminate SHS, efforts are needed to educate the public about the dangers of SHS exposure and to promote the voluntary adoption of smoke-free rules in private settings, particularly among subpopulations at greatest risk of exposure. In addition, jurisdictions with comprehensive policies prohibiting smoking in public places and worksites, including restaurants and bars, could extend protection from SHS to areas that are typically exempted from these policies, including multiunit housing and vehicles occupied by young people.

## References

[1] US Department of Health and Human Services. The health consequences of involuntary exposure to tobacco smoke: a report of the Surgeon General. Atlanta (GA): Centers for Disease Control and Prevention, National Center for Chronic Disease Prevention and Health Promotion, Office on Smoking and Health; 2006. 20669524

[2] Centers for Disease Control and Prevention. Smoking-attributable mortality, years of potential life lost, and productivity losses — United States, 2000–2004. MMWR Morb Mortal Wkly Rep 2008;57(45):1226–8. 19008791

[3] US Department of Health and Human Services. How tobacco smoke causes disease: the biology and behavioral basis for smoking-attributable disease: a report of the Surgeon General. Atlanta (GA): Centers for Disease Control and Prevention, National Center for Chronic Disease Prevention and Health Promotion, Office on Smoking and Health; 2010. 21452462

[4] Centers for Disease Control and Prevention. State Activities Tracking and Evaluation (STATE) System. http://apps.nccd.cdc.gov/statesystem/Default/Default.aspx. Accessed February 18, 2013.

[5] Goodman PG , Haw S , Kabir A , Clancy L . Are there health benefits associated with comprehensive smoke-free laws? Int J Public Health 2009;54(6):367–78. 10.1007/s00038-009-0089-8 19882106

[6] Haw SJ , Gruer L . Changes in exposure of adult non-smokers to secondhand smoke after implementation of smoke-free legislation in Scotland: national cross sectional survey. BMJ 2007;335(7619):549. 10.1136/bmj.39315.670208.47 17827485 PMC1976488

[7] Centers for Disease Control and Prevention. Vital signs: nonsmokers’ exposure to secondhand smoke — United States, 1999–2008. MMWR Morb Mortal Wkly Rep 2010;59(35):1141–6. 20829748

[8] Giovino GA , Chaloupka FJ , Hartman AM , Gerlach Joyce K , Chriqui J , Orleans CT , Cigarette smoking prevalence and policies in the 50 states: an era of change — the Robert Wood Johnson Foundation ImpacTeen tobacco chart book. http://www.impacteen.org/statetobaccodata/chartbook_final060409.pdf. Accessed February 18, 2013.

[9] Centers for Disease Control and Prevention. Disparities in secondhand smoke exposure — United States, 1988–1994 and 1999–2004. MMWR Morb Mortal Wkly Rep 2008;57(27):744–7. 18614993

[10] Centers for Disease Control and Prevention. State-specific prevalence of smoke-free home rules — United States, 1992–2003. MMWR Morb Mortal Wkly Rep 2007;56(20):501–4. 17522588

[11] Centers for Disease Control and Prevention. State-specific secondhand smoke exposure and current cigarette smoking among adults — United States, 2008. MMWR Morb Mortal Wkly Rep 2009;58(44):1232–5. 19910910

[12] Cartmell KB , Miner C , Carpenter MJ , Vitoc CS , Biggers S , Onicescu G , Secondhand smoke exposure in young people and parental rules against smoking at home and in the car. Public Health Rep 2011;126(4):575–82. 21800752 10.1177/003335491112600414 PMC3115217

[13] Hitchman SC , Fong GT , Borland R , Hyland A . Predictors of smoking in cars with nonsmokers: findings from the 2007 Wave of the International Tobacco Control Four Country Survey. Nicotine Tob Res 2010;12(4):374–80. 10.1093/ntr/ntq008 20156887 PMC2847075

[14] King BA , Dube SR , Tynan MA . Current tobacco use among adults in the United States: findings from the National Adult Tobacco Survey. Am J Public Health 2012;102(11):e93-100. 10.2105/AJPH.2012.301002 22994278 PMC3477973

[15] Council of American Survey Research Organizations. (1997). The rules we live by: code of standard and ethics for survey research. http://www.scribd.com/doc/17276719/Code-of-Standards-and-Ethics-for-Survey-Research-CASRO. Accessed February 18, 2013.

[16] Centers for Disease Control and Prevention. State smoke-free laws for worksites, restaurants, and bars — United States, 2000–2010. MMWR Morb Mortal Wkly Rep 2011;60(15):472–5. 21508923

[17] Centers for Disease Control and Prevention. Vital signs: current cigarette smoking among adults aged ≥18 years — United States, 2005–2010. MMWR Morb Mortal Wkly Rep 2011;60(35):1207–12. 21900875

[18] Cheng KW , Glantz SA , Lightwood JM . Association between smoke-free laws and voluntary smoke-free home rules. Am J Prev Med 2011;41(6):566–72. 10.1016/j.amepre.2011.08.014 22099232 PMC3222862

[19] Fong GT , Hyland A , Borland R , Hammond D , Hastings G , McNeill A , Reductions in tobacco smoke pollution and increases in support for smoke-free public places following the implementation of comprehensive smoke-free workplace legislation in the Republic of Ireland: findings from the ITC Ireland/UK Survey. Tob Control 2006;15(Suppl 3):iii51–8. 10.1136/tc.2005.013649 16754947 PMC2593063

[20] Osypuk TL , Acevedo-Garcia D . Support for smoke-free policies: a nationwide analysis of immigrants, US-born, and other demographic groups. Am J Public Health 2010;100(1):171–81. 10.2105/AJPH.2009.160218 19910345 PMC2791266

[21] Siahpush M , McNeill A , Hammond D , Fong GT . Socioeconomic and country variations in knowledge of health risks of tobacco smoking and toxic constituents of smoke: results from the 2002 International Tobacco Control (ITC) Four Country Survey. Tob Control 2006;15(Suppl 3):iii65–70. 10.1136/tc.2005.013276 16754949 PMC2593062

[22] US Environmental Protection Agency. Smoke-free homes. http://www.epa.gov/smokefree/. Accessed June 15, 2012. 11645578

[23] Fiore MC , Jaen CR , Baker TB , Bailey WC, Benowitz NL, Curry SJ, Clinical practice guideline. Treating tobacco use and dependence: 2008 update. Rockville (MD): US Department of Health and Human Services, Public Health Service; 2008. http://www.ncbi.nlm.nih.gov/books/NBK63952/. Accessed June 15, 2012.

[24] Van Deusen A , Hyland A , Travers MJ , Wang C , Higbee C , King BA , Secondhand smoke and particulate matter exposure in the home. Nicotine Tob Res 2009;11(6):635–41. 10.1093/ntr/ntp018 19351784

[25] Jones MR , Navas-Acien A , Yuan J , Breysse PN . Secondhand tobacco smoke concentrations in motor vehicles: a pilot study. Tob Control 2009;18(5):399–404. 10.1136/tc.2009.029942 19706642

[26] Mills AL , Messer K , Gilpin EA , Pierce JP . The effect of smoke-free homes on adult smoking behavior: a review. Nicotine Tob Res 2009;11(10):1131–41. 10.1093/ntr/ntp122 19633273

[27] Centers for Disease Control and Prevention. Healthy homes manual: smoke-free policies in multiunit housing. http://www.cdc.gov/healthyhomes/Healthy_Homes_Manual_WEB.pdf. Accessed November 8, 2011.

[28] The Center for Social Gerontology. Smoke-Free Environments Law Project. Housing authorities/commissions which have adopted smoke-free policies. http://www.tcsg.org/sfelp/SFHousingAuthorities.pdf. Accessed November 8, 2011.

[29] Global Advisors on Smokefree Policy (GASP). Smoke-free vehicles when children are present. http://www.njgasp.org/f_SF%20cars,kids,%20info,%20arguments.pdf. Accessed November 8, 2011.

[30] Max W , Sung HY , Shi Y . Who is exposed to secondhand smoke? Self-reported and serum cotinine measured exposure in the U.S., 1999–2006. Int J Environ Res Public Health 2009;6(5):1633–48. 10.3390/ijerph6051633 19543411 PMC2697933

